# A virtual reality simulation of a novel way to illuminate the surgical field – A feasibility study on the use of automated lighting systems in the operating theatre

**DOI:** 10.3389/fsurg.2023.1055053

**Published:** 2023-03-02

**Authors:** Timur Cetin, Andre Mühlenbrock, Gabriel Zachmann, Verena Weber, Dirk Weyhe, Verena Uslar

**Affiliations:** ^1^ University Hospital for Visceral Surgery, Pius Hospital Oldenburg, University of Oldenburg, Oldenburg, Germany; ^2^ Centre for Computer Graphics and Virtual Reality, University of Bremen, Bremen, Germany

**Keywords:** virtual reality, surgical lighting, virtual reality simulation, smart operating room, EEG, mental effort, smart lighting

## Abstract

**Introduction:**

Surgical lighting systems have to be re-adjusted manually during surgery by the medical personnel. While some authors suggest that interaction with a surgical lighting system in the operating room might be a distractor, others support the idea that manual interaction with the surgical lighting system is a hygiene problem as pathogens might be present on the handle. In any case, it seems desirable to develop a novel approach to surgical lighting that minimizes the need for manual interaction during a surgical procedure.

**Methodes:**

We investigated the effect of manual interaction with a classical surgical lighting system and simulated a proposed novel design of a surgical lighting system in a virtual reality environment with respect to performance accuracy as well as cognitive load (measured by electroencephalographical recordings).

**Results:**

We found that manual interaction with the surgical lights has no effect on the quality of performance, yet for the price of a higher mental effort, possibly leading to faster fatigue of the medical personnel in the long run.

**Discussion:**

Our proposed novel surgical lighting system negates the need for manual interaction and leads to a performance quality comparable to the classical lighting system, yet with less mental load for the surgical personnel.

## 1. Introduction

The operating room (OR) is a challenging place to work. Sometimes extremely complex surgical procedures, e.g., transplantations or large surgeries of the internal organs of the abdomen, have to be conducted in a complex setting. While those surgeries naturally require a lot of skill and knowledge from the surgeons conducting the procedure, the work has to be conducted in a team-setting that might include team members that are new to the field (i.e., students, nurses in training or doctors learning new techniques) or who have secondary tasks beside the surgery that might lead to phone calls etc. conducted in the OR setting. The head surgeon has to be able to work in this environment with his/her full concentration on the procedure ahead while leading the team in a responsible way to keep all team members motivated to fulfill their tasks to the best of their abilities while under a high amount of time (and thereby financial) pressure to keep up with the hospitals pre-scheduled operating plan.

Naturally, over the last years many technical improvements have been made to ease the surgeons work pressure, mainly with the aim of allowing for quicker and safer surgeries to be conducted. Quick surgeries benefit not only the organizational side of the hospital as schedules can be arranged more tightly, thus allowing for more procedures in one day do be conducted, but they will also benefit the patient as time under anesthesia can be kept as brief as possible. These technical advances in the operating room can be found especially in the well-equipped theaters of large medical centers or medical colleges in the developed world and will include, but are not limited to robotic surgery to improve the techniques of surgery itself, telemedicine that improves the availability of specialized personnel if the need arises during a procedure or artificial intelligence (AI) supported diagnostic methods. All these methods are believed to make the OR personnel's work easier, allowing them to concentrate better on the work ahead – thereby increasing patients safety indirectly – or increase patients safety directly, e.g., with the better availability of specialized personnel or diagnostic techniques (1–5).

Sufficient illumination is an essential part of the work environment in an operating room (OR) or, as a matter of fact, in any other work-related task that puts high demands on accurate performance in strenuous situations. It is a necessary requirement for good performance by the medical team and thereby essential for patient safety (6). However, considering the technical advances surgical and OR technology made over the last serval decades, and especially in the last several years, the question of proper illumination in the OR seems to be often overlooked.

While multiple approaches to additional illumination in the OR have been made – e.g., in the development of special head-mounted lights or surgical microscopes – the main source of illumination across virtually all surgical disciplines in the OR currently still is the overhead lamp, designated as the Surgical Lighting System (SLS) (7). While SLSs are actualy a rather complex systems reflecting the need for focused, bright illumination of the surgical field in the context of proper, general ambient illumination in the OR itself, for many personnel that is working with the SLS on a daily basis, it is often not considered anything more than a bright lamp (7). All of those standard SLS that are available today require the personnel to manually interact with the lights, i.e., to adjust the lights manually by using a handle on the lamp. This handle might either be autoclaved or covered in a sterile plastic cover to allow the surgeon to handle the lamp directly or to ask for re-positioning of the light's focus by other personnel. Considering that studies have shown that OR-staff re-adjust the SLSs manually on average every 7.5 min during a procedure, a concern for contamination may be raised as studies by Schweitzer et al. indicate the presence of microorganisms (including pathogens) in about half of all supposedly sterile SLS handles (8, 9). Additional concerns might include the induction of burns on the patient or even fires in the operating theatre. Even though this is more a concern when head-mounted lights are used rather than overhead SLSs, case reports of overhead SLSs causing patients' burns are known (7). As currently classical SLS with incandescent lights are being phased out and LED technology has become standard for SLS, the ideas of thermal damage due to improperly used SLS has become less of a concern, but considering only these two potential problems with the traditional SLS alone, the need for modernized illuminating systems in the OR becomes clear (10, 11).

This need becomes even more evident when the complex issue of human-machine-interaction is additionally considered. In this context the interaction with the SLS may have the potential to (partially) distract the user from the primary task, i.e., conducting the surgery. It is generally accepted that SLS may be a part of these distractors, e.g., as a result of equipment malfunction or even simply through the need for manual interaction with the SLS, e.g., by re-adjustment during an operative procedure (8). Even if a surgeon might not subjectively report the interactions with the SLS while performing surgery as an additional stressor, a sub-conscious, possibly automated mental process has to take place anyways. While the need for re-adjustment of the SLS has to be identified, the handle has to be griped and the SLS has to be brought to its new, desired position. This task seems trivial, but it adds another sensorimotor coordination task to an environment that already puts great strain on one's mental capabilities, requiring complex visuo-motor-coordination even without the added burden of interacting with the SLS. Considering this underlying psychological mechanism, it seems important that the mental toll of interaction between surgeon and SLS is further investigated (8).

The study presented here is part of the German Federal Ministry of Education and Research's SmartOT project that aims at overcoming those shortcomings of the currently used SLS. Within the SmartOT project, a real-life prototype of a novel kind of SLS is to be developed and implemented for testing in a mock surgical theatre to determine its benefit for every-day use. The proposed novel SLS will implement light sensors to detect whether or not the surgical field is properly illuminated and, if not, automatically adjusts the lights in a way that no manual interaction by the surgical staff is required, thus eliminating a potential source of infection for the patient as well as presumably freeing mental capacity for the surgeon. To test the feasibility of this proposed design and to estimate how much of a benefit for the quality of work by the surgical personnel this proposed design is, a virtual reality (VR) simulation of the proposed prototype was used on a limited number of participants while neuropsychological testing (to determine the possible increase in work quality when no need to interact with the SLS arises) as well as electroencephalographic (EEG)-recordings (to determine the possible decrease in mental/cognitive load when no need to interact with the SLS arises) was conducted.

VR simulations have become more-and-more sophisticated over the past years and are currently the state-of-the-art way to display computer graphics. In-line with the technical improvement of VR simulations, high-end computers and VR headsets have become increasingly affordable and reliable, so naturally VR applications have recently become a more common part of medicine. Still, the use of VR technology can largely be separated into two distinct groups: either VR is used as part of medical training, e.g., to familiarize students with examination techniques or doctors with new methods, or VR is used with a patient-centered focus to help inform patients (e.g., about an upcoming surgical procedure) or as part of therapy (e.g., in psychiatric illnesses or during psychological treatment) (12–17). We are not aware of any approach so far using VR to explore the ergonomic benefits of the medical work environment, e.g., when planning a new OR or setting up new technical appliances in a pre-defined space. One of the aims of our study is to show the possible benefits of testing novel approaches from an ergonomic point-of-view prior to constructing and testing elaborate and expensive real-life set-ups.

## 2. Materials and methods

The proposed design of this novel SLS consists a computer controlling an array of multiple, small lights in the OR's ceiling. Three sensors situated above the OR table register the situation below constantly and give real-time feedback to the computer to make sure that the surgical site is constantly illuminated. If shadows are cast onto the surgical site or any other obstruction occurs (e.g., due to the position of the personnel in the OR), this is registered by the sensors and the individual lamps of the array are activated or deactivated and aligned in such a way that the surgical site is again completely illuminated.

During the tests, the participants' EEG was recorded, both in real-life as well as in the VR simulation. This allowed for comparison of the task-related neurophysiological effects of a similar task under both conditions, thus giving an idea of the reliability of VR simulations when compared to the actual real-life task. We hypothesize that, under the condition in which a manual interaction with the SLS is required, participants will show a worse performance in the neuropsychological D2-test as well as a higher cognitive load in the EEG compared to the condition with a static SLS and that this is seen in the real-life application as well as in the VR simulation. This investigation is not merely of academic interest but might have significant impact on the practical approach to surgical lighting given that the state of the development of so-called *smart technologies* has made a significant step towards its practical usability in the last years.

One of the aims of our study is investigating how strong the possible distraction of the OR personnel by interaction with the SLS is on the level of performance as well as on the level of psychological burden, measured as EEG-activity, which we see as a corelate of the mental activity/cognitive load. To investigate these effects and its possible solution by implementing smart technologies, we conducted two studies with two distinct experimental conditions and different groups of participants. In the first part of our investigation, subjects conducted the D2 test of attention once with and once without the need to adjust the SLS during the testing (18, 19). This approach gave us an indication of the general effects of interaction with the SLS. In the second part of our investigation, we simulated the manual interaction with a classical SLS as well as the proposed novel approach to the smart surgical lighting in a VR-simulation during the same neuropsychological test of attention as in the real-life testing. This approach gave us an impression if the novel approach to SLS may be a benefit to the surgical staff. Altogether, four conditions were investigated: (a) Testing under constantly illuminated conditions in a real-life setting, (b) Testing under conditions of manually re-adjusting the SLS in a real-life setting, (c) Testing with automatically re-adjusting lights in a VR simulation and finally (d) Testing under conditions of manually re-adjusting the SLS in a VR simulation. During all evaluations, the test performance is obtained as a parameter of quantifying the effect of manual interaction with the SLS on the accuracy of the subjects work as well as the EEG to gain an impression mental load in the current condition.

All testing took place in university of Oldenburg's the department of visceral surgery, located at PIUS hospital Oldenburg.

### 2.1. Participants

Participants (with or without prior experience of interaction with SLS) were recruited from the Universities of Oldenburg (Germany) and Bremen (Germany) or from the staff of the PIUS hospital in Oldenburg (Germany). All participants participated voluntary in the study and received no financial or other compensation. The experiments were approved by the University of Oldenburg's medical ethical committee (Local File-No. 2020-146) and written consent was obtained from each participant prior to the experiment. All participants were asked for possible exclusion criteria (neurological or psychiatric disorder, left-handedness, psychoactive medication) prior to the first session.

No participants took part in both arms of the study, i.e., participants either took part in the real-life or in the VR-testing. This was done to include possible learning/memory effects on the D2-test.

### 2.2. Neuropsychological testing

Participants had to complete the revised version of the *D2 test of visual and sustained attention*, either in the pen-and-paper version during the real-life testing or in the digital version during the VR-testing. This test is a well-established test for selective and sustained attention in a time-critical visual screening task that has been established as a standard neuropsychological tool (19). Completing one set of the D2-test takes 280 s, during which participants are asked to screen a line of two different letters (d & *p*) from the left to the right. Using either a pen or pencil (or a touch with the controller/click with the mouse during the VR version), participants have to mark the target stimuli, a *d* that is surrounded by two dashes either below and/or above the letter. All other letters as well as the *d* marked with more or fewer dashes than two are to be ignored. After 20 s, the participants are instructed to proceed to the next line. This procedure is finished after the participant has completed 14 lines. The results are evaluated for the amount of correctly identified target items as well as for the wrongfully marked non-target items, the amount of missed target items and the ratio between those.

### 2.3. Electrophysiological recordings

Mobile EEG recordings [sampling rate 250 Hz, *smarting mobi* (mbt, Belgrade, Serbia)] with 24 electrodes (Fp1, Fp2, AFz, F7, F3, Fz, F4, F8, T7, C3, Cz, C4, T8, CPz, M1, M2, P7, P3, Pz, P4, P8, POz, O1, O2) were obtained with a standard *EasyCap* EEG-recording cap (Easycap, Herrsching, Germany) and stored in.xdf-format before processed for analysis. EEG were analyzed using EEGlab (V2020.0, open source toolbox for Matlab R2020a MathWorks, Natick, Massachusetts, United States) on a standard PC running the Windows 10 operating system (Microsoft, Redmond, Washington, United States).

### 2.4. Analysis

After completion of the session, the D2-Test was analyzed as instructed by the test's manual for the number of items processed and the accuracy of the test, defined by the number of missed target items. Descriptive statistical analysis was done for the results of the D2-Test by calculating the mean values and standard deviation of the processed target items for the whole test sheet as well as only for the lines, during which manipulation occurred. The mean number of errors, i.e., missed target items, was also calculated for the whole test. The mean percentages and standard deviation of missed target items was calculated as a measurement of participants accuracy.

The EEG was filtered with a low pass threshold at 3 Hz and a high pass threshold at 30 Hz and then visually inspected for artefacts and general technical quality before further analysis. As adjusting the SLS during testing took up to 5 s, the first 5 s of the EEG for each experimental line was excluded from analysis due to exclude any possible motor artefacts from manual interaction with the lamp. The remaining EEG was subjected to frequency analysis, both for the first two seconds after interaction with the SLS and for the whole remaining 15 s period after the interaction with the SLS. Data was compared for the lines in which the SLS was manipulated between the experimental condition and the corresponding lines during control condition. For this, EEG was subjected to frequency analysis to show activity in alpha, beta and theta frequency bands.

#### 2.4.1. Statistical analysis

Differences between the conditions for both, EEG-data (alpha-band at 8 Hz and beta-band at 27 Hz at all electrode positions) as well as performance in the D2-test (no. of items processed and number of mistakes made) were determined by using a paired t-test (SPSS V27, IBM, Armonk, New York, United States) for the behavioral as well as for electrophysiological data. The 8 Hz and 27 Hz points were chosen as the those were the frequencies of peak activity for the alpha as well as beta band. Comparison was done by t-test only for the group “manual adjustment” vs. the group “static lamp” during the real-life testing as well as for the group “manual adjustment” vs. the group “automated lamp” in the VR testing, but not across the arms of the study, i.e., no t-test comparison was done between data from the VR arm of the study vs. data from the real-life arm of the study as both arms had different groups of participants.

Additional to t-test testing, 2 × 2 factorial ANOVAs were conducted for EEG data, for the peak alpha band activity at 8 Hz as well as for the peak beta band activity at 27 Hz across all 4 groups (manual SLS adjusting in real life, static SLS in real life, manual adjusting SLS in VR, automatically adjusting SLS in VR).

### 2.5. Experimental protocol – *real-life testing*

9 participants (5 male, 4 female, mean age 32 years ± 8.1 years) participated in two sessions each, using a state-of-the-art SLS (*Dr Mach LED 3*, Ebersberg, Germany). In one session (control condition), the participant performed the pen-and-paper D2-Test (D2-R Test, Hogrefe, Göttingen, Germany) on an examination couch in one of the surgical examination rooms of the hospitals surgical department. While the room was dark, the only source of light was the SLS positioned in such a way over the examination couch that only the test sheet was sufficiently illuminated while the participant conducted the test. During the other session (experimental condition), the conditions were likewise with the exception of the SLS being moved in a defined way several times during the testing, thus requiring the participant to manually re-adjust the light while conducting the D2-test at the same time. EEG data was recorded during both sessions. To exclude any possible learning effect that might influence the D2-Test, control- and experimental condition were done alternately, i.e., while the first participant started with a control condition, the second participant would start with the experimental condition and vice versa. At the sessions start, participants were instructed on how to complete the D2-Test using the standard manual provided with the test (19). After instruction, the D2-Test was conducted. During the experimental condition, the SLSs focus of light was moved about 40 cm–50 cm to the right side by the investigator in 6 out of 14 lines (at the beginning of the lines 3, 5, 6, 8, 11 and 12) of the D2-test, thus requiring the participant to re-adjust the SLS in such a way that the D2-Test sheet was illuminated again. As per default in the D2 Test the first and last line are not included in the analysis, the SLS needed to be re-adjusted in half of the lines later included in the analysis. Both conditions were investigated with an interval of about one week between them.

### 2.6. Experimental protocol – virtual reality testing

**Figure 1:**
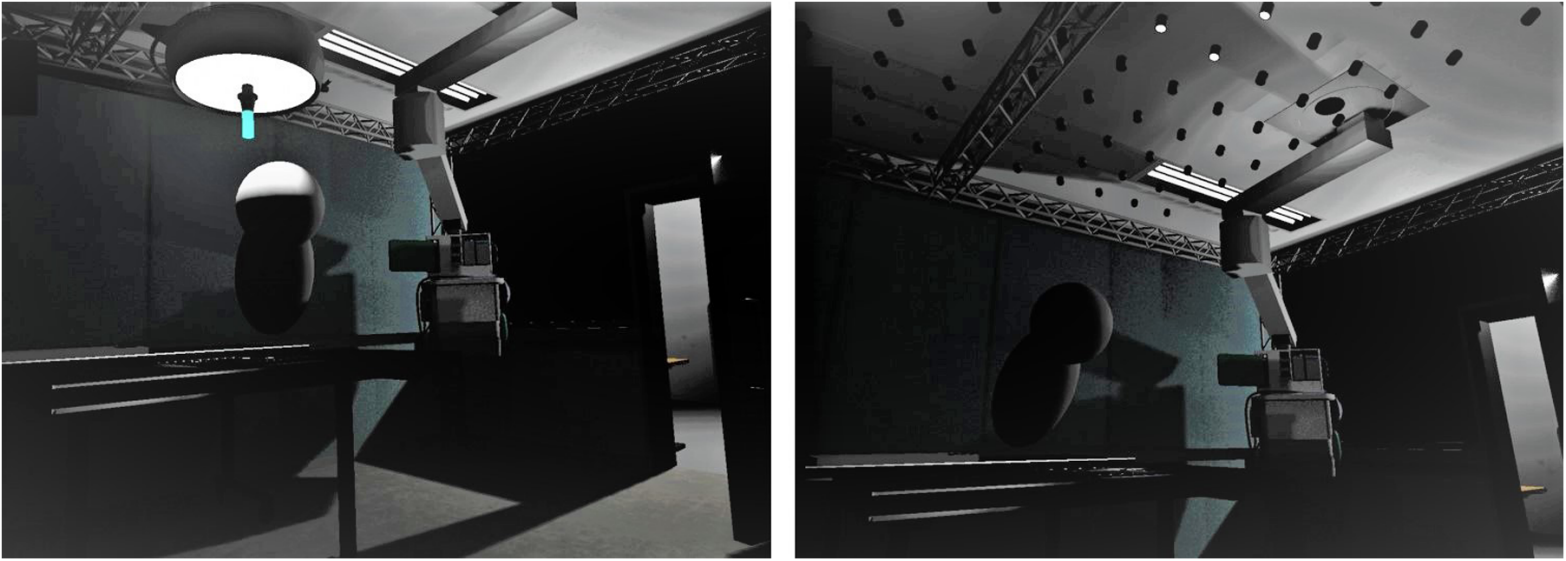
The VR-environment showing a dark OR-like room with only the table illuminated by the SLS. Once light from the SLS is blocked from reaching the table, e.g., by the occluder in the centre above the table, the working area appears dark and light needs to be re-adjusted by grabbing the green handle of the SLS using a VR-controller (left) or adjusts automatically with the novel SLS (right).

**Figure 2:**
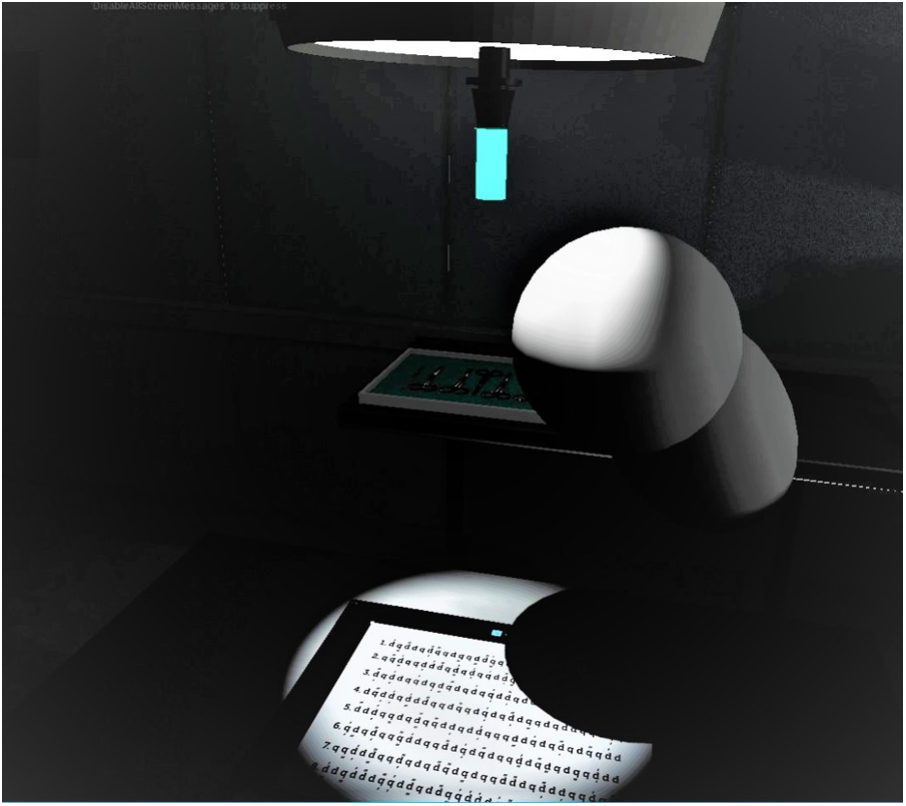
The digital version of the D2-test is shown on the simulated operating table, partially concealed by an occluder roughly resembling the shape of a human. In this situation, the light source simulated to be above the table needs to be re-adjusted manually.

**Figure 3:**
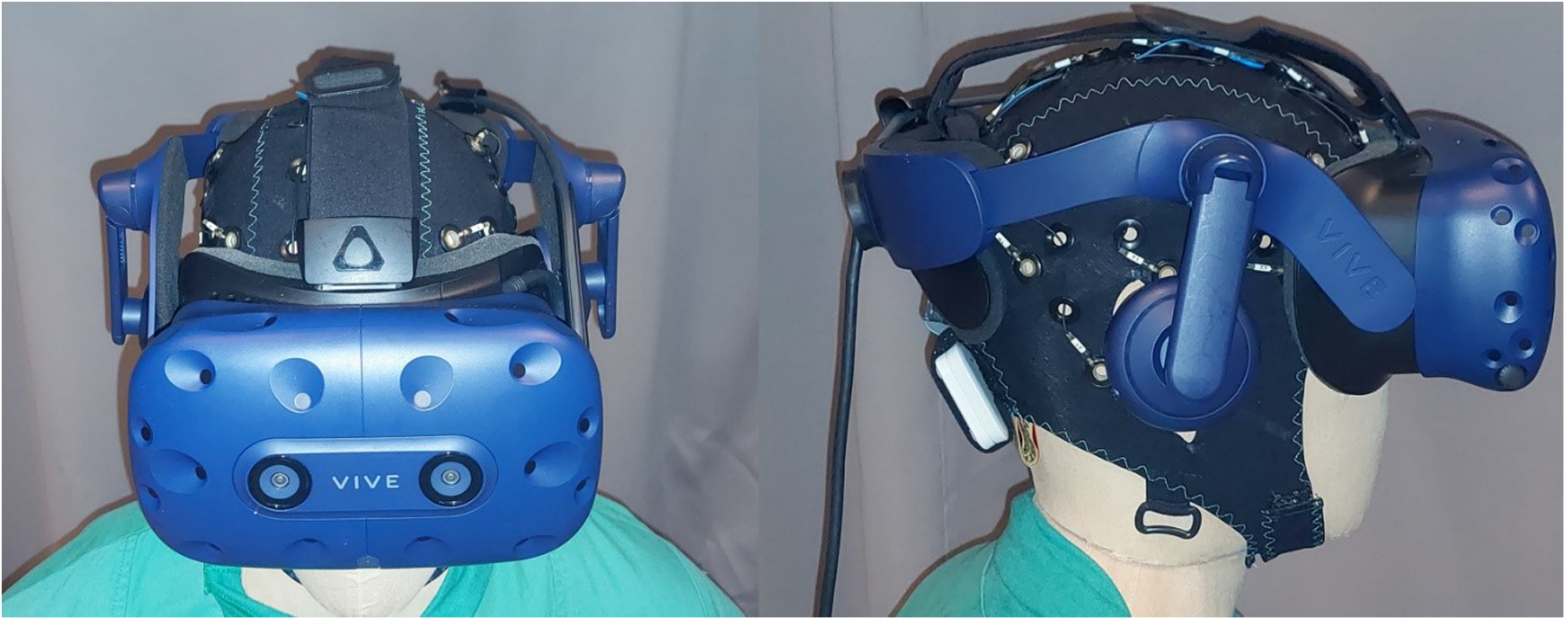
Experimental set-up for VR-testing: VR-headset combined with EasyCap recording cap and mobile EEG-device.

**Figure 4:**
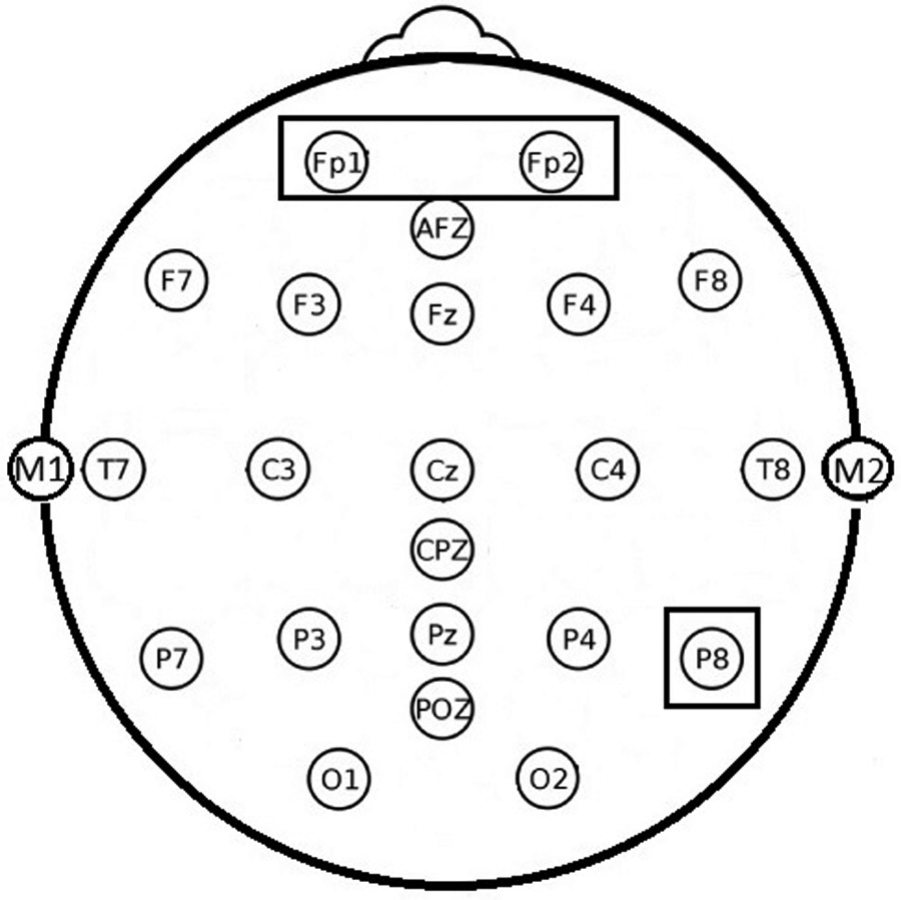
Electrode positions used for EEG recordings. Note the highlighted positions Fp1, Fp2 and P8 were experimental effect were found.

Similar to real-life testing, participants conducted a comparable task in a VR-simulated environment. This was done to simulate more closely an OR-like situation as well as the implementation of a system that is comparable to the proposed, novel SLS. 8 participants (4 male, 4 female, mean age 26 years ± 10.4 years) participated in two sessions each, using a state-of-the-art virtual reality headset (HTC Vive pro, HTC, Taoyuan, Taiwan). The VR scenario was presented on a computer running the Windows 10 operating system (Microsoft, Redmond, Washington, United States) using the unreal engine (EpicGames, Raleigh, North Carolina, United States) to present a OR-like scenario created by the center for computer graphics and virtual reality (CGVR) from the university of Bremen (Germany). Figure 1 illustrates this scenario: From the point-of-view of the participant, the operating table can be seen in the center-left of the room. The room is kept dark, only illuminated by the SLS: on the left side a classical SLS with a green handle that can be griped and hold by using a VR controller to be handled like a real-life SLS, on the right the same VR environment with the novel SLS with multiple small light in the ORs ceiling, here with three single light units being active. On the opposite site of the operating table an occluder can be see: A body roughly resembling the shape of a human (Figure 1). In this setting, the participant had the impression of standing in front of an operating table with the digital version of the D2 test of attention being shown on the operating table instead of the surgical site. To mark the target item, analogue to the paper version, the items had to be touched with one of the controllers, that was represented by a pen in the VR simulation (Figure 2). During the sessions, analogue to the pen-and-paper testing, at the start of lines 3, 5, 6, 8, 11 and 12, the need to adjust the light was evoked by having an occluder, i.e., a shape roughly resembling a human body, appearing above the operating table, blocking the light from reaching the operating table by casting a shadow. This scenario resembles the situation in the OR more closely than the real-life testing as it simulates a situation where a member of the surgical team blocks the light by casting a shadow in the operating field. If this occurred during the control condition, the light adjusted automatically in a way that it would also when the novel SLS is implemented, thus not requiring any action from the participant. During control condition on the other hand, the participants had to adjust the SLS manually once the light was occluded from reaching the operating table. Therefore, a classical SLS was simulated in the VR environment. The handle of the simulated SLS had to be griped using the VR controller and the SLS had to be re-adjusted in such a way that the operating table was illuminated again, thus once again resembling the situation in the real-life testing situation. EEG data was recorded during both sessions while the VR-Headset was worn over the EEG-Cap (Figure 3). Recordings were done at standard electrode locations (Figure 4). To exclude any possible learning effect that might influence the D2-Test, also control- and experimental condition were done alternately, i.e., while the first participant started with a control condition, the second participant would start with the experimental condition and vice versa. At the sessions start, participants were instructed on how to complete the D2-Test using the standard introduction and practice steps that are shown at the start of each digital D2-test. After instruction, the D2-Test was conducted. Both conditions were investigated with an interval of about one week between them.

## 3. Results

### 3.1. Behavioural testing

With the D2-Test, participants performed a test that is considered to require a high amount of concentration while under a moderate, but persistent amount of time-related stress. We believe that this was true for our approach, even though no direct evidence for the mental demands of the D2-Test can be drawn from our study. We have conducted the NASA-TLX test of task loads in relation to the D2-Test in pilot testings for our study and have found participants to rate the mental demands while conducting the D2-Test as 11/20, the temporal demands during the D2-Test as 13/20 and the overall situational stress during the D2-Test as 12/20. This indicates a moderate, but subsisting level of mental demands caused by the D2-Test of attention.

**Figure 5:**
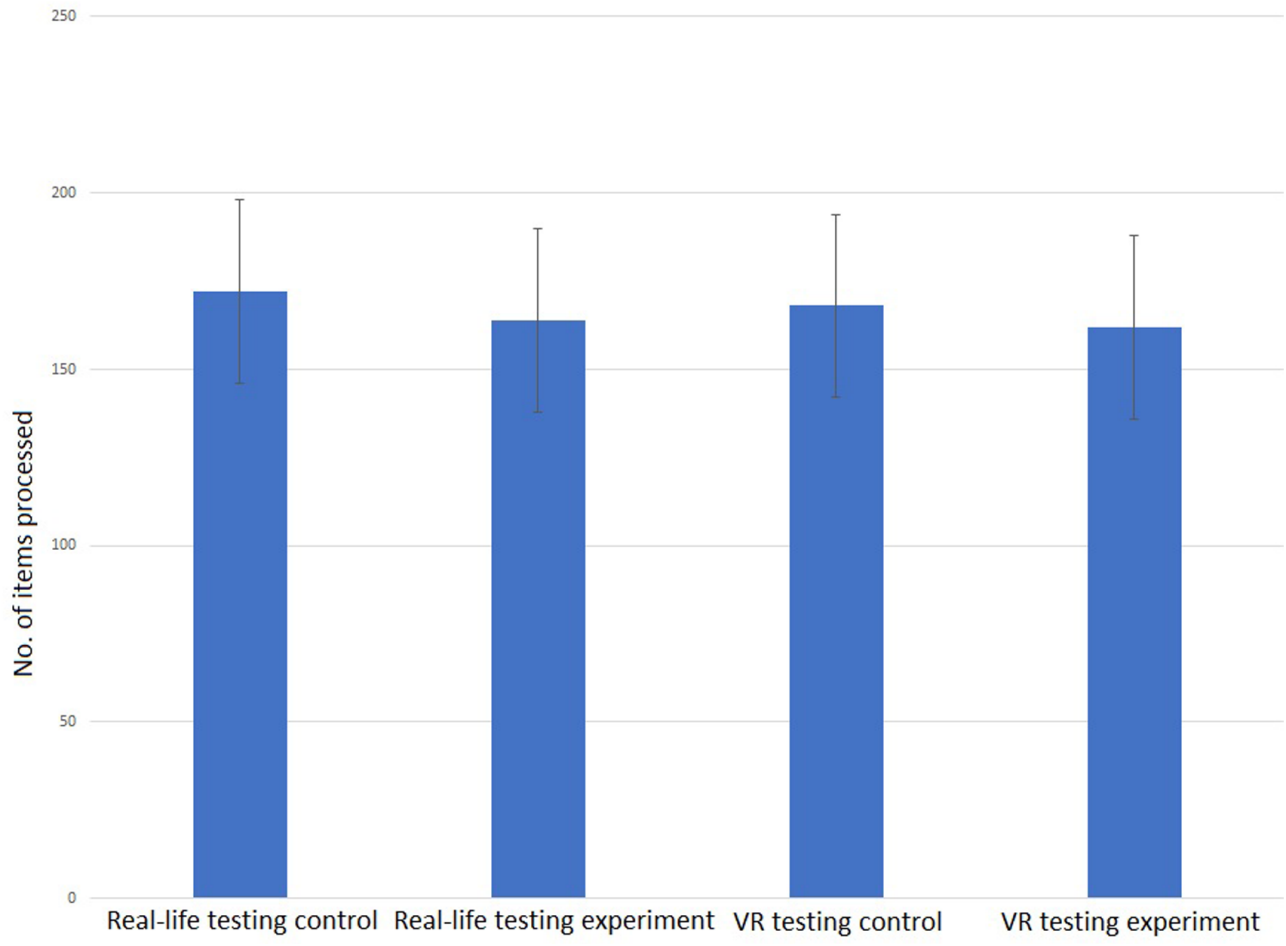
Mean No. of items processed during the whole D2-behaviourial test in control and experimental condition (real-life testing left [172 items, SD 26 vs. 164 items, SD 25, *t*(8) = 2.17, *p* = 0.062]; VR-testing right [168 items, SD 18 vs. 162 items, SD 14, *t*(7) = 1.97, *p* = 0.081]. No statistically significant differences between the conditions could be found.

**Figure 6:**
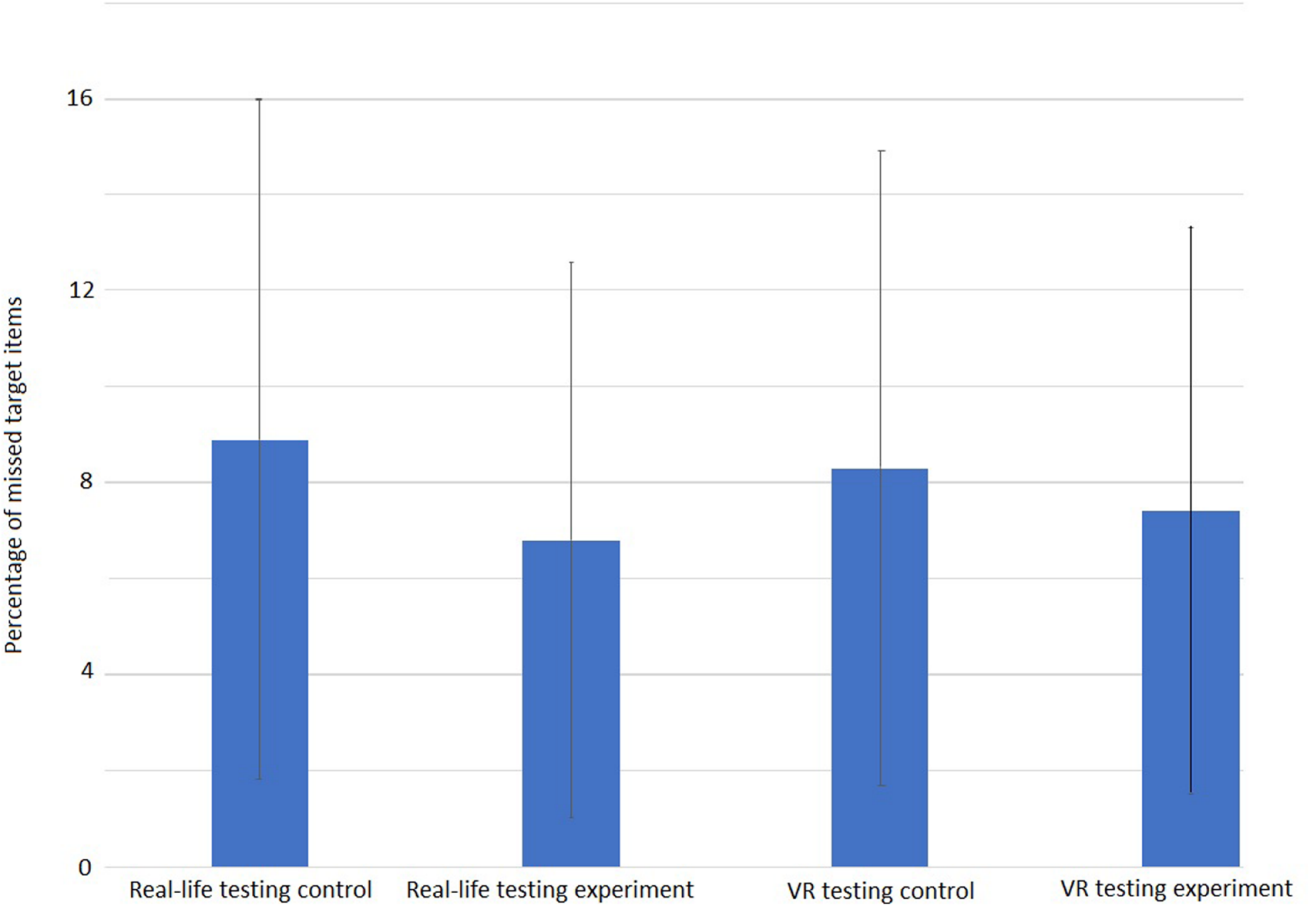
Average accuracy of performance in the D2-test of attention shown as percentage of missed target items. In Real-Life testing (left) 8.9%, under control conditions (SD 7.1) vs. 6.8% (SD 5.8) during experimental conditions while in the VR-Testing, under control conditions 8.3% of target items were missed (SD 6.6) vs. 7.4% (SD 5.9) under experimental conditions. No statistically significant difference between conditions was found (Real-Life: *t*(8) = 1.052; *p* = 0.324, VR: *t*(7) = 1.42; *p* = 0.449).

Between the participants performance in the D2-Test in control and experimental condition, no differences were found. If anything, a slight tendency towards better performance under experimental condition was observed:

Comparing the number of processed items, no difference between the conditions was seen on the level of the overall test as well as for only the lines in which the SLS was manipulated. On the overall level, an average number of 172 items was processed in the real-life testing during the control condition vs. 164 items during the experimental condition (SD ±26 items vs. ±25 Items) while during the VR testing, an average overall of 168 items was processed in the control condition and of 162 items in the experimental condition (SD ±18 items vs. ±24 Items) (Figure 5). Considering only the lines of the D2-test in which the SLS was actually manipulated, also no statistically significant difference was found for either real-life or VR testing. The amount of missed target items does not differ whether the SLS was adjusted during the test or not. If anything, there is a slight tendency towards less target items being missed under experimental conditions that can be seen in the performance accuracy of the participants, i.e., the relation of missed target items to overall processed items. While under control conditions in the real-life testing, an average of 8.9% of the target items were missed by the participant (SD ±7.1%), under experimental conditions only an average of 6.8% target items were missed (SD ±5.8%). During the VR testing, similar results had been found with an average of 8.3% (SD ±6.6%) of the target items missed in the control condition and 7.4% during the experimental condition (SD ±5.9%) (Figure 6). Large variations between the participants reflect a large individual variation with some participants even showing a higher number of items processed when the SLS had to be adjusted. Even though the difference in the mean number of overall processed items does not reach a level of statistical significance when compared between the conditions, it might still be considered a small tendency towards more items being processed during the experimental condition.

### 3.2. Electrophysiology

In the participants' EEG, small but persistent differences were found in alpha and beta activity on the first 2 s after adjusting the SLS. No effects were found on the 15 s scale as well as on the theta band for both time scales.

**Figure 7:**
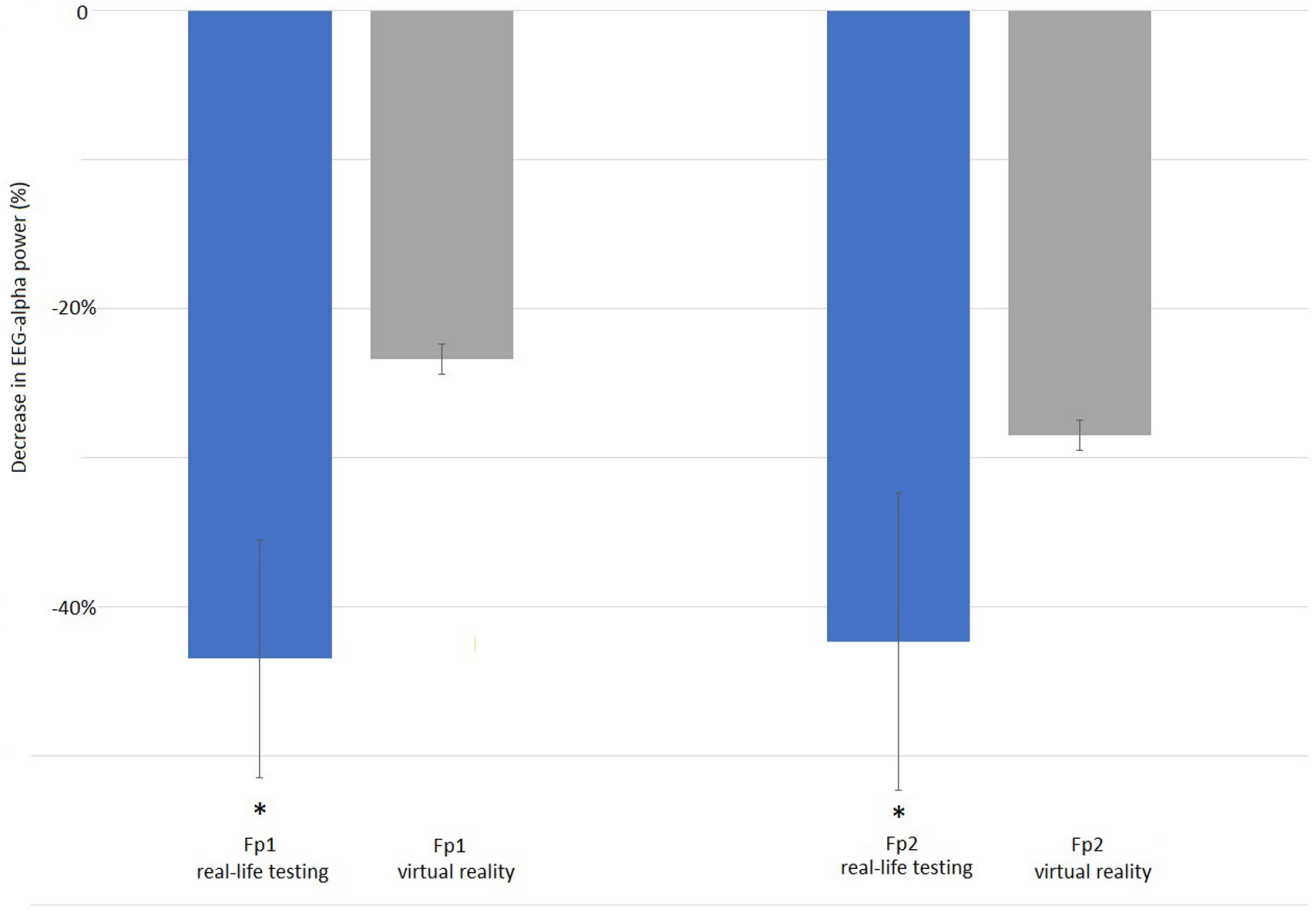
Reduction of alpha-band activity at electrode positions Fp1 (left group) and Fp2 (right group): EEG alpha-band activity at 8** **Hz is significantly reduced at position Fp1 by −43.5% during manually handling the SLS in real-life testing [left blue bar, SD 8, *t*(6) = 10.69, *p* = 0.002] when compared to the automated SLS. Also at position Fp2 the reduction in alpha-band activity is significant with −42.35% when testing is done in real-life [SD 10, *t*(6) = 8.74, *p* = 0.003]. During virtual reality testing (grey bars), the decrease in alpha EEG power at positions Fp1 (left grey bar) and Fp2 (right grey bar) is also present with −23.4% (SD 19) at Fp1 and −28.5% (SD 16) at Fp2, but does not reach the level of statistical significance [Fp1: *t*(7) = 4.83, *p* = 0.371], *t*(7) = 3.74, *p* = 0.453).

**Figure 8:**
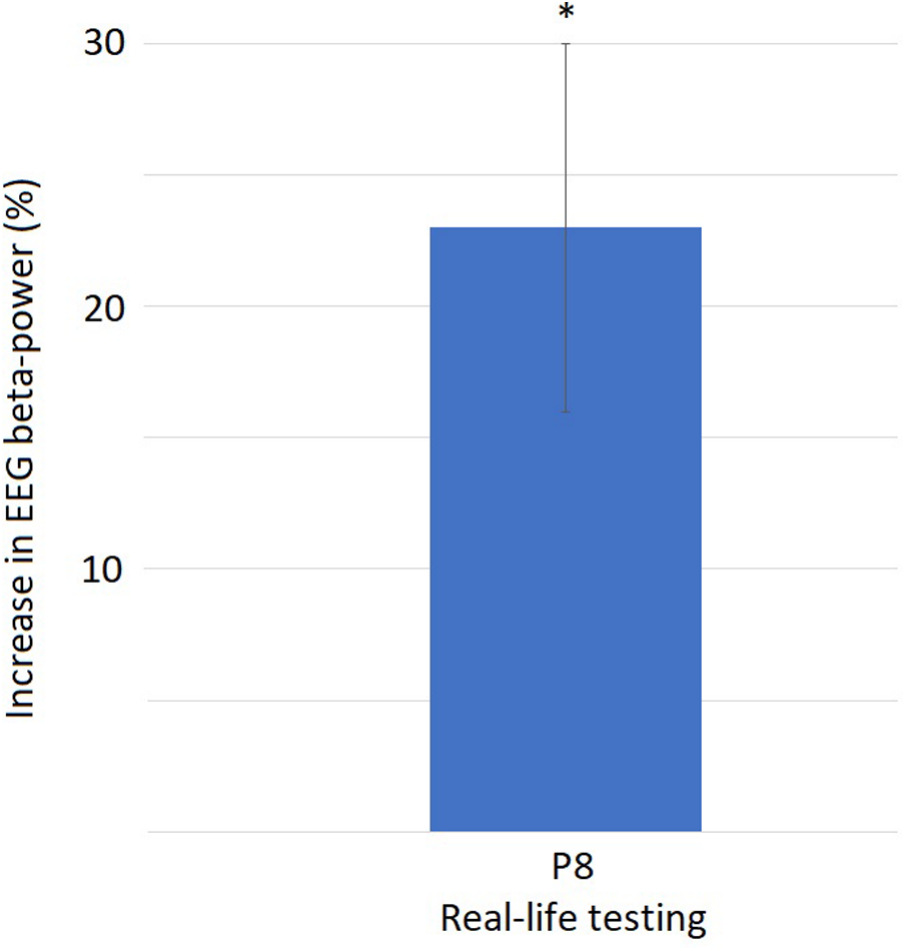
Increase of beta-band activity at electrode position P8: EEG beta-band activity at 27 Hz increases significantly by 23% [SD 7, *t*(6) = 3.92, *p* = 0.002] when manual interaction with the SLS is required compared to the automated SLS.

By t-test-testing, in the alpha band, significantly decreased alpha power in frontal positions Fp1 and Fp2 could be seen in the real-life testing. Compared to control condition, EEG power in the alpha band decreased during experimental condition in both fronto-polar positions with the largest effect in the low alpha band at 8 Hz by more than 40%. In VR testing, a tendency towards a decreased alpha was also found in the frontopolar positions without reaching the level of statistical significance (Figure 7). Additionally, for the real-life testing, we find a significantly increased power by 23% over parietal position P8 with the largest effect in the high beta band at 27 Hz (Figure 8).

## 4. Discussion

The OR is a very challenging work environment. On top of the actual conduction of the surgery, multiple factors are adding to the mental workload. In a practical setting, these different components cannot be separated from each other, i.e., the interaction with the SLS is only one factor adding to the workload, together with factors like social interaction, background noise in the OR etc. To ease the burden of workload on the surgeon, it seems important to identify the amount that each of these single factors adds to the overall workload. This is only possible by finding a laboratory condition that allows for investigating these factors in a one-by-one approach. In our case the interaction with the SLS was investigated in a setting that is semi comparable to that in an OR but excludes all stressful factors except the interaction with the SLS. Our expectation was to find that the interaction with the SLS influences the performance on a basic level, i.e., time lost due to adjusting the SLS leads to a worse performance as fewer items could be processed. This hypothesis was not confirmed, but after closer analysis of the data it was found that the interaction with the SLS seems to influence the surgeon's performance indeed. When interpreting the results of the statistical testing, it should be pointed out that the number of participants from which data was collected is small. Interpreting data from only 8 or 9 participants in this study allows for getting a general indication how the use of the novel SLS might influence work performance and mental load and how well this novel SLS can be tested prior to actual set-up of a real-life prototype in an VR simulation, but also lead to a large standard deviation and consequently only limited reliability of any indicators of statistical significance.

### 4.1. Behavioral testing

The number of processed items did not differ between experimental and control condition. This might be explained by a well-known a mechanism of compensation: Performance reached in undisturbed control conditions might reflect the genuine performance of an individual, i.e., the individual's threshold. If the conditions change in an unfavorable way by making the completion of the task more difficult, the individual unconsciously puts increased mental effort into the task ahead, leading to the same performance as under control conditions. This effect was found in the real-life testing as well as in the VR-approach. This is not unexpected as the digital version of the D2 test of sustained attention has been validated and shows comparable results to its pen-and-paper counterpart and the real-life experimental setup is comparable to the VR. Finding the same effect under both conditions indicates to a robust psychological effect. This can be seen as both, a validation of our finding that interaction with the SLS leads to no worsening of performance as well as another validation of the comparability of the digital version of the D2 test with its original, pen-and-paper version. As out study is the first time the D2 test of visual attention has been conducted in a VR setting our results show that the D2-test is as valid if presented in a VR setting as it is when presented on a computer screen in the standard digital version.

It has long been known that mental resources are allocated dynamically during a complex task, meaning that there is constant feedback about performance and re-allocation of mental resources during completing a task. This takes place as maintaining attention is an energy consuming effort, leading to attention always being maintained in the most energy efficient way possible for the present situation (20). Changing the situation in an unfavorable way causes mental resources to be newly adapted to the requirements of the task ahead, allocating more mental resources to the primary task when a secondary task has to be completed simultaneously – in our case completing the D2-test as a primary task while adjusting the SLS as the secondary task. This maintenance of good performance in the primary task leads to increased mental effort, thus causing faster fatigue (21). This effect has been reported for other complex tasks, e.g., driving a car (22). We speculate that we observe a similar pattern in our study: driving a car as a primary task might be comparable in its complexity to completing the D2-test (or even conducting surgery for a trained professional), and compensatory mechanisms may be taking place when adjusting the SLS (secondary task) thus showing that there is an influence of interaction with the SLS on cognitive and mental load. Still, we can only speculate that such mechanisms are taking place by transferring what literature indicates for conducting primary and secondary tasks while driving a car to the work environment, i.e., in our case the OR (24). To our knowledge, there are no studies investigating the mental burden when adjusting a SLS as a secondary task while conducting surgery as the primary task, so no literature can provide a robust backing for such a speculation.

### 4.2. Electrophysiology

EEG-data supports the idea that the effect of maintaining the original performance level is achieved at the cost of a higher mental load. The fact, that the EEG effects were only found during the first 2 s after adjusting the SLS and not in the overall EEG indicates that the effects can be attributed to the prior interaction with the SLS.

It can be expected to find the basic motor action of interacting with the SLS reflected in the EEG. This would be represented in an increased activation of electrodes located above the motor cortex, e.g., at parietal positions. For the beta band activity on parietal position P8, we found an increased activity for the real-life testing only. Finding an increased beta activity over the motor cortex at electrode position P8 during the experimental condition compared to control might indicate that this increase is simply due to the increased motor activation needed in the experimental condition. This occurring only in the real-life condition might be simply attributed to the higher motor effort of physically handling and adjusting with a certain physical force the actual SLS vs. simply using a VR controller. However, this explanation falls short of explaining why the increase in beta activity was found over the same (right) side as the motor action was conducted with. If the beta increase would have been solely due to motor action, it would have been expected to be on the side contralateral to the motor action only, i.e., over the left hemisphere as the motor action was conducted with the right hand. The EEGs beta band is somewhat poorly understood, but seems to play a role in multiple processes. Beside roles in working memory as well as stimulus perception, classification and response, an important role of beta oscillations seems to lie in sensor-motor responses as well as in attention (24, 25). Literature gives some indication that beta oscillations play a role in directing attention towards a visual stimulus, possibly reflecting the increased visual attention needed when the mental effort has to be increased during the experimental condition. This would be in line with already published data (26, 27) and could indicate that the explanation for the beta increase in P8 might be rooted in a more complex, neuropsychological process, which should be investigated in further analysis and studies.

EEG power in the alpha band is affected in frontal positions in both, the real-life as well as the VR testing. This effect is larger in the real-life testing, were alpha decreases reach the level of statistical significance at both locations, Fp1 and Fp2, while there is only a tendency without reaching the level of statistical significance in the VR testing. On both electrode locations, alpha EEG power is decreased with the largest effect in the low alpha band at 8 Hz. Alpha activity in the EEG has long been investigated and was found to correlate with multiple psychological and neurological functions (28). One of the most common and most widely accepted roles for alpha activity is seen in attention-related mechanisms. Alpha activity is seen as the rhythm of the resting brain that is increasingly inhibited with increased attention, i.e., the fewer alpha power is found, the more active the brain is, and the more attention is dispatched to the task ahead, and the higher is the cognitive and mental load (29). As we find less alpha activity during the experimental condition, the experimental task requires more attention and has thereby a higher mental load then the control condition. The fact that this effect is observed at Fp1 and Fp2 further supports the idea of observing an effect that is part of a rather complex, highly cognitive process, since these electrode positions record activity originating in the orbitofrontal cortex that is generally attributed with higher cognitive functions. Most likely the decreased alpha activity in frontopolar positions can be attributed to the complex procedure of identification of the need to adjust the SLS, briefly dispatching attention to this task, initiating and consecutively executing the task of adjusting the SLS while paying attention to finding the right position of the light and subsequently re-adjusting the attention to the primary task. This finding may not be too surprising, still underlines our idea that interaction with the SLS requires a significantly higher mental effort and thereby might increase mental fatigue. It is unclear why this effect reaches the level of statistical significance only in the real-life testing but not in the VR-testing. One possible explanation might simply be due to an overall decreased EEG quality due to mechanical artefacts of interaction between the VR headset and EEG electrodes, that can never be excluded to compromise the quality of EEG-recording when a VR-Headset is used in combination with a sensitive EEG-cap. As no increased artefacts were seen during visual EEG-inspection, it seems more likely than the differences are due to physiological and/psychological reasons. Even though the VR simulation used a scan of an actual OR, the VR simulation might not have been immersive enough to simulate the real OR in a complete way. Especially the manual interaction with the SLS might have been less immersive in the VR setting than it is in real life: When manually adjusting the SLS, a certain mechanical force is needed and mechanical (haptic) feedback is given. Studies have shown that the handling forces needed to adjust the SLS manually might be quite considerable and influence the way the personnel adjusts the lamp (30). As in our simulation, only a VR controller was used to simulate the handle of a SLS, but no haptic feedback in form of manual counter-force was given, this might be considered a lack of immersion resulting in a less pronounced effect in the simulation vs. real-life testing.

Using the 2 × 2 factorial ANOVA for additional statistical testing, no additional statistical significance has been found between the groups. This is not unexpected as the number of participants in this study was small and individual variances were large, resulting in a large standard deviation, thus giving generally little power to any statistical outcome. The absence of additional statistical differences indicates to us, that the EEG recordings in general are reliable as no difference between VR and real-life testing has been found.

Overall, our study indicates that the interaction with the SLS in an OR is a factor in adding mental burden on the OR staff. The effect seems to be small but robust, and does not seem to be a relevant factor in practical means for short medical interventions as adverse effects can be well compensated by normal, psychophysiological processes. However, these compensatory mechanisms seem to put additional strain on the mental capabilities of the surgeon, being one of the factors leading to faster mental fatigue, compared to a condition were no interaction with the SLS is required. Faster fatigue may in consequence lead to worse performance in surgical procedures where high mental alertness is required for a longer period of time, in consequence indicating that the implementation of the proposed automated SLS is beneficial for maintaining the highest possible level of patient safety. These results underline the need for novel SLS that eliminates the need for manual interaction.

## 5. Conclusion

We have simulated our proposed, novel SLS design on a limited number of participants only, thus giving little power to our statistical testing. Still, this is not the main focus of our approach which should be seen more as a “proof of principle” as to how a new, hands-free design of a future SLS might look like and as how well a neuroergonomic question can be addressed by simulating a medical work environment in VR. We understand that every SLS that allows for the surgeon to deliver the same quality of work as a classical SLS but without the need to interact manually would be a great benefit for the patient, as a possible source of infection is eliminated. We can see from our results, that our approach might be a feasible way of archiving this and, moreover, that we can successfully conduct a first testing of such a set-up in a VR simulation before constructing the real-life prototype.

We have conducted a study that, for the first time, gives an impression of the scale of the effect that is caused by interaction with SLS on mental capabilities and performance in the OR. We see that the effect is small, but persistent over the conditions. The small magnitude of the effect makes us believe that a hands-free SLS will be of benefit mainly for hygiene reasons in every-day work in the OR, but can also be a factor of easing the surgeons mental load when it comes to procedures lasting long, i.e., several hours.

We conclude, that the establishment of a touch-free SLS seems to be desirable not only from the hygienic point of view, but also to decrease the mental burden on the surgical staff. The current approach of implementing smart technologies to minimize the need of manual interaction might thus prove to be a feasible way of overcoming the shortcoming of the current SLS and those approaches may successfully be tested in a VR simulation prior to implementation in real-life.

## Data Availability

The raw data supporting the conclusions of this article will be made available by the authors, without undue reservation.
